# Stemness is Derived from Thyroid Cancer Cells

**DOI:** 10.3389/fendo.2014.00114

**Published:** 2014-07-15

**Authors:** Risheng Ma, Simon Bonnefond, Syed A. Morshed, Rauf Latif, Terry F. Davies

**Affiliations:** ^1^Thyroid Research Unit, Department of Medicine, Icahn School of Medicine at Mount Sinai and the James J Peters VA Medical Center, New York, NY, USA; ^2^Reims University of Medicine, Reims, France

**Keywords:** epithelial–mesenchymal transition, cancer stem cells, thyroid papillary carcinoma, celastrol, thyroid peroxidase, thyroglobulin, Braf

## Abstract

**Background:** One hypothesis for thyroid cancer development is its derivation from thyroid cancer stem cells (CSCs). Such cells could arise via different paths including from mutated resident stem cells within the thyroid gland or via epithelial to mesenchymal transition (EMT) from malignant cells since EMT is known to confer stem-like characteristics. Furthermore, EMT is a critical process for epithelial tumor progression, local invasion, and metastasis formation. In addition, stemness provides cells with therapeutic resistance and is the likely cause of tumor recurrence. However, the relevance of EMT and stemness in thyroid cancer progression has not been extensively studied.

**Methods:** To examine the status of stemness in thyroid papillary cancer, we employed a murine model of thyroid papillary carcinoma and examined the expression of stemness and EMT using qPCR and histochemistry in mice with a thyroid-specific knock-in of oncogenic Braf (LSL-Braf(^V600E^)/TPO-Cre). This construct is only activated at the time of thyroid peroxidase (TPO) expression in differentiating thyroid cells and cannot be activated by undifferentiated stem cells, which do not express TPO.

**Results:** There was decreased expression of thyroid-specific genes such as Tg and NIS and increased expression of stemness markers, such as Oct4, Rex1, CD15, and Sox2 in the thyroid carcinoma tissue from 6-week-old BRAF^V600E^ mice indicating the dedifferentiated status of the cells and the fact that stemness was derived in this model from differentiated thyroid cells. The decreased expression of the epithelial marker E-cadherin and increased EMT regulators including Snail, Slug, and TGF-β1 and TGF-β3, and the mesenchymal marker vimentin demonstrated the simultaneous progression of EMT and the CSC-like phenotype. Stemness was also found in a cancer thyroid cell line (named Marca cells) derived from one of the murine tumors. In this cell line, we also found that overexpression of Snail caused up-regulation of vimentin expression and up-regulation of stemness markers Oct4, Rex1, and CD15, with enhanced migration ability of the cells. We also showed that TGF-β1 was able to induce Snail and vimentin expression in the Marca cell thyroid cancer line, indicating the induction of EMT in these cells, and this induction of EMT and stemness was significantly inhibited by celastro a natural inhibitor of neoplastic cells.

**Conclusion:** Our findings support our earlier hypothesis that stemness in thyroid cancer is derived via EMT rather than from resident thyroid stem cells. In mice with a thyroid-specific knock-in of oncogenic Braf (LSL-Braf(^V600E^)/TPO-Cre), the neoplastic changes were dependent on thyroid cell differentiation and the onset of stemness must have been derived from differentiated thyroid epithelial cells. Furthermore, celastrol suppressed TGF-β1 induced EMT in thyroid cancer cells and may have therapeutic potential.

## Introduction

The role of stem cells in thyroid cancer development remains unclear (1). There is no doubt that the thyroid gland retains a significant number of resident stem cells as shown in mice and human thyroid tissue (2–5) but the role of thyroid cancer stem cells (CSCs) in tumor formation and progression is less certain (1, 6). There are two major possibilities. The first is that neoplastic transformation of a normal resident thyroid stem cell takes place and leads to tumor formation during which the stem cells undergo asymmetric divisions to secure their own survival as we have previously demonstrated (7). The second possibility is that stemness in thyroid cancer is derived directly from malignant thyroid epithelial cells via epithelial to mesenchymal transition (EMT). This is a well-known biologic process defined by the loss of epithelial-specific characteristics, the acquisition of a fibroblast-like morphology, reduced cellular adhesion, and increased motility during embryonic development (8). Cells undergoing EMT develop stem cell-like features such as the ability to self-renew (9). From a molecular point of view, EMT is characterized by the loss of epithelial markers, including E-cadherin and cytokeratins and the gain of mesenchymal-like gene expression program, such as fibronectin, vimentin, and N-cadherin (8). The use of a mouse model in which the malignant transformation can only occur in differentiated thyroid cells expressing thyroid peroxidase (TPO) is a simple approach to answering the question of where stemness is derived from in thyroid cancer. Our data point clearly to the important role of EMT in providing stem-like characteristics in papillary thyroid cancer.

Epithelial to mesenchymal transition is also a commonly accepted key pathologic mechanism in epithelial tumor progression and has been well shown to allow acquisition of stem-like properties by cancer cells (8, 10, 11). The connection between the loss of E-cadherin and the gain of vimentin expression by cancer cells has been established by many studies (12) and has been associated with the development of invasive cancer, metastatic dissemination, and poor clinical prognosis in various human tumors (8) including thyroid carcinomas (13, 14). Several distinct mechanisms of E-cadherin down-regulation and vimentin up-regulation have been described. In particular, transforming growth factor-β (TGF-β) (including TGF-β1, TGF-β2, and TGF-β3 isoforms) has been shown to regulate the expression of genes that play key roles in a large variety of biological phenomena, ranging from tissue remodeling to tumor initiation and progression (15). TGF-β1 induced overexpression of all stemness genes tested including Oct4, Nanog, Sox2, and CD133 and caused loss of epithelial morphology, assumption of a fibroblast-like appearance, and up-regulation of vimentin, Slug, Twist and down-regulation of cytokeratin and E-cadherin (16). Several zinc-finger transcriptional factors, such as Snail (17, 18), Slug (19), or Twist (20), serve as downstream effectors of the TGFβ pathway and have been shown to induce the EMT through direct or indirect suppression of E-cadherin and up-regulation of vimentin transcription in cancer and thus contribute to tumor cell growth, migration, and invasion (8, 21).

In this report, we studied thyroid papillary cancer in mice with a thyroid-specific knock-in of oncogenic Braf [LSL-Braf(^V600E^)/TPO-Cre] (22) in order to determine if stemness was present in such tumors and whether EMT was the source of such characteristics.

## Materials and Methods

### Mice

BrafV600E mice with a thyroid-specific knock-in of BrafV600E were established by crossing LSL-BrafV600E mice, in which a latent Braf mutant knock-in allele can be activated by Cre recombinase through excision of a floxed STOP cassette (23), with TPO-Cre mice, which express Cre under the control of the human TPO promoter (24), which is active only in thyroid follicular cells beginning at E14.5. Mice were in mixed genetic backgrounds. Both LSL-BrafV600E mice and TPO-Cre mice were kindly provided by Dr. James A. Fagin (Memorial Sloan-Kettering Cancer Center). All procedures were approved by the Institutional Animal Care Committee of Icahn School of Medicine at Mount Sinai.

### Marca cells – a Braf^V600E^ cancer cell line

Primary tumors of Braf^V600E^ mice were minced and resuspended in RPMI 1640 with 10% fetal bovine serum (FBS) containing 100 U/ml type I collagenase (Sigma) and 1 U/ml dispase (Roche). Enzymatic digestion was carried out for 45 min at 37°C. Then, cells were seeded in RPMI 1640 supplied with 10% FBS, 100 IU penicillin/ml, and 100 μg/ml streptomycin. After growing to confluence, cells were passaged. The doubling time of this line was 35.75 h and the cells retained the Braf mutation as shown by PCR genotyping of DNA isolated from WT and heterozygous LSL-BrafV600E (without Cre) thyroids and the Marca cells (data not shown).

### Plasmids and transduction

The pCMV6-Snail vector was electroporated into thyroid cancer cells (Neon transfection system; Invitrogen Life Technologies), and after 2 days, the cells were cultured in G418 (0.8 mg/mL) for 4 weeks until resistant clones were established. High-expressing clones were chosen for expansion into stable lines.

### Cell proliferation and viability assays

Cells were plated in 96-well plates, incubated for 24 h at 37°C, and treated with TGF-β1 (R&D), 0.5 μM Celastrol (Sigma) for 48 h. Proliferation of cells was measured using a Cell Counting Kit-8 (Dojindo) according to the manufacturer’s protocol.

### Differentiation of thyroid CSCs into adipocytes

Marca and MarcaSnail cells were cultured in medium alone or medium supplemented with 10 μg/mL transferrin, 0.5 mg/mLinsulin, 0.2 nM T3, 1 μM dexamethasone, and 1 mU/mL TSH for up to 15 days. Cells were then collected for analysis.

### RNA isolation and RT-PCR

Total RNA was extracted from BRAF^V600E^ mice thyroid tumor tissues and normal thyroid gland, and cultured human thyroid cancer cells using the RNeasy system (Qiagen Ltd). cDNA synthesis was performed using the SuperScript III system (Invitrogen Corp.). Quantitative (q)RT-PCR was performed by using the SYBER Green PCR system on a StepOnePlus instrument (Applied Biosystems). Relative expression levels of each RT-PCR product was analyzed using the 2^−ΔΔCT^ method and normalized to the expression of the housekeeping gene GAPDH. Data presented (mean) are from three independent experiments in which all sample sets were analyzed in triplicate.

### Immunohistochemistry

Paraffin-embedded, formalin-fixed tumor tissues from Braf^V600E^ mice and normal thyroid gland from same strain mice were sectioned into 6 μm thick slices. Sections were deparaffinized in xylene and rehydrated in a series of graded ethanol, and their antigens were retrieved by heating the slides in a citrate buffer (pH 6.0). Endogenous peroxidase activity was quenched with 0.3% H_2_O_2_ in methanol for 10 min at room temperature. The sections were then incubated for 20 min with 2.5% normal house blocking serum. This procedure was followed by incubation with the primary antibodies at proper dilution overnight at 4°C. Negative controls were obtained by replacing the primary antibodies with control isotype IgG. Staining was visualized using the ImmPRESS™ reagent (Vector Laboratories, Burlingame, CA, USA) detection system with peroxidase substrate solution. Slides were counterstained in hematoxylin and coverslipped in synthetic media.

### Wound-healing assay

Cells were plated in six-well culture plates in complete culture medium and grown to 90% confluence. A wound was made by scrapping with a sterilized 100 μl pipette tip in the middle of the cell monolayer. Cells were then cultured with fresh complete culture medium containing 5 ng/ml TGFβ with or without 0.5 μM celastrol treatment for 24 h. After that, the ability of cells to migrate into the cleared section was observed and photographed.

## Results

### Expression of thyroid-specific genes and stem cell markers

**Figure 1:**
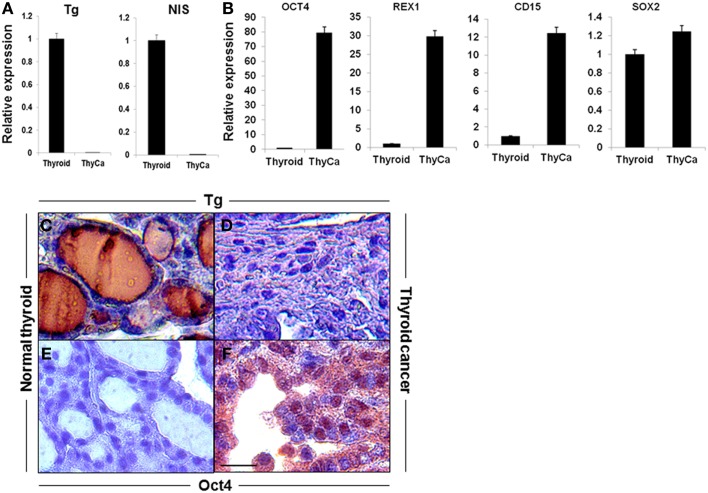
**Expression of thyroid-specific genes and stem cell markers in mouse thyroid and thyroid tumor**. **(A,B)** Expression of thyroid-specific genes **(A)** and stem cell markers **(B)** in mouse normal thyroid gland and BRAF^V600E^ mice thyroid tumors as measured by qRT-PCR. The data were normalized using GAPDH as an endogenous control. The results are expressed as mean ± SEM of three independent experiments. **(C–F)** Representative images of immunostaining of Tg **(C,D)** and OCT4 **(E,F)** in normal thyroid gland **(C,E)** and thyroid tumor tissue **(D,F)**.

qRT-PCR analysis were performed for thyroid-specific markers in total mRNA from 6-week old BRAF^V600E^ murine thyroid tumors and wild type normal thyroid. Expression of Tg and NIS was markedly decreased in the tumor tissue (Figure 1A), while the stem cell markers (Oct4, Rex1, CD15, and Sox2), although detectable in normal thyroid, were greatly increased in the neoplastic tissues (Figure 1B). These data indicated the undifferentiated state of the BRAF^V600E^ tumor specimens and indicated the presence of cells with CSC-like features. These results were confirmed by immunohistochemical analysis of Tg, which was barely detectable in the tumor tissue while Oct4 was overexpressed (Figures 1C–F).

### Expression of EMT markers in thyroid cancer

**Figure 2:**
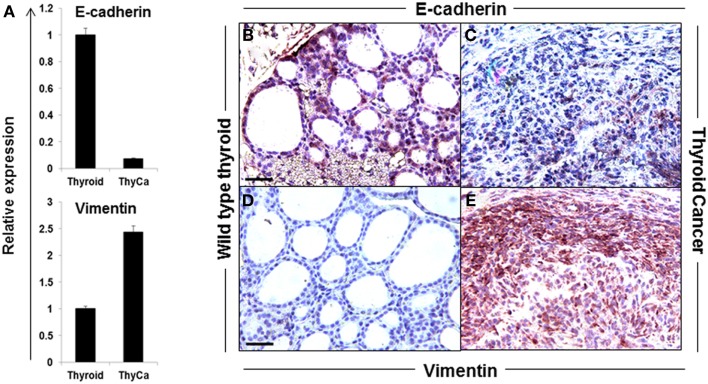
**Expression of EMT markers in normal mouse thyroid and thyroid tumor**. **(A)** Expression of E-cadherin and vimentin in normal mouse thyroid and BRAF^V600E^ mice thyroid tumor by qRT-PCR. The data were normalized using GAPDH as an endogenous control. The results are expressed as mean ± SEM of three independent experiments with three parallels. **(B–E)** Representative images of immunostaining of E-cadherin **(B,C)** and vimentin **(D,E)** in normal mouse thyroid **(B,D)** and thyroid tumor **(C,E)**.

The hallmark of EMT is the down-regulation of E-cadherin and the up-regulation of vimentin expression. A significant loss of E-cadherin gene expression and an increase in vimentin gene expression was seen in the BRAF^V600E^ thyroid tumors compared to normal thyroid (Figure 2A) and similar data were obtained by immunohistochemistry where vimentin staining was highly positive only in BRAF^V600E^ tumor specimens (Figures 2B–E). Morphologically, thyroid cancer cells differed from the polarized, epithelial shape of the wild type thyroid cells, and appeared as a spindle-shaped phenotype (Figures 2C,E). The data demonstrated that while E-cadherin was well expressed in normal thyroid tissue, its expression was markedly decreased and vimentin expression up-regulated in the thyroid carcinomas from the transgenic mice over-expressing BRAF^V600E^ (25). Such information supported the transition of thyroid cells into undifferentiated CSC-like cells by passing through EMT (9, 26).

### Expression of EMT inducers in thyroid cancer

**Figure 3:**
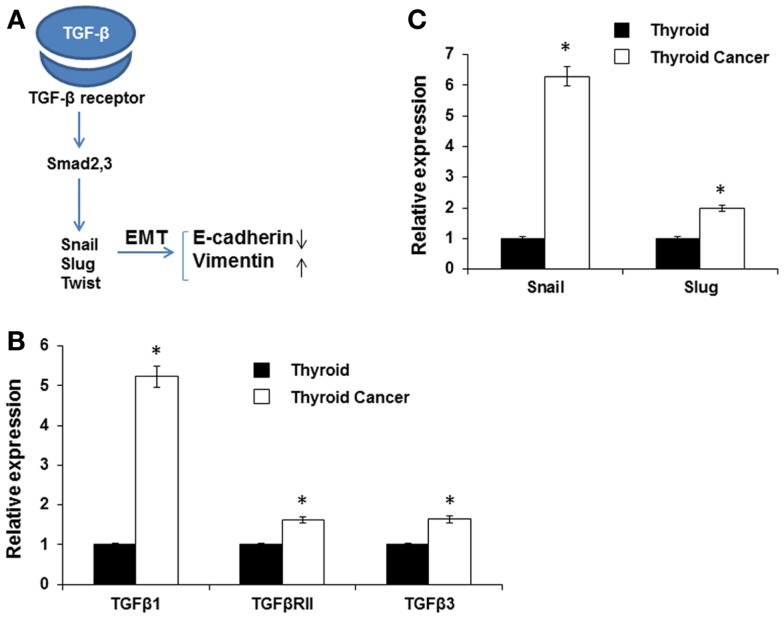
**Expression of EMT inducers**. **(A)** Schematic illustration of EMT signaling. **(B,C)** Expression of TGF-β1, TGF-β2R, and TGF-β3 **(B)**, and SNAIL and SLUG **(C)** in normal mouse thyroid and thyroid tumor by qRT-PCR. The data were normalized using GAPDH as an endogenous control. The results are expressed as mean ± SEM of three independent experiments with three parallels. *p < 0.05.

TGF-β is a multifunctional cytokine that plays a dual role in cancer; in early stages it inhibits tumor growth, whereas later it promotes invasion and metastasis formation (27–29). TGF-β is also a known major inducer of EMT and its pro-invasive action is thought to be via EMT induction of a series of transcriptional repressors including Snail, Slug, and Twist (28, 30) (Figure 3A). We found that TGF-β, Snail, and Slug were highly expressed in the murine thyroid cancer tissue (Figures 3B,C). TGF-β binds to TGF-βRII that subsequently phosphorylates the TGF-βRI activin-receptor like kinase 5, which in turn phosphorylates Smad2 and Smad3 (28). Then the complex of Smad proteins translocates into the nucleus where it is able to interact with the Snail and Slug transcription factors and jointly regulate target genes, inducing down-regulation of the epithelial marker E-cadherin and up-regulation of the mesenchymal marker vimentin (31). We found that the expression of TGF-β1, TGF-β2R, and TGF-β3 were all increased in BRAF^V600E^ mice thyroid tumors compared to wild type normal thyroid tissue (Figure 3B).

Expression of Snail has been detected in many different types of primary human cancers, including breast, colon, and stomach cancer (32). Here, we observed that expression of both Snail (sixfold) and Slug (twofold) was increased in BRAF^V600E^ murine thyroid tumor tissue compared to normal thyroid (Figure 3C). The results demonstrated an inverse relationship between E-cadherin and TGF-β1 or Snail expression (33) consistent with TGF-β1 contributing to the induction of EMT in thyroid cancer via the Snail family of transcription factors.

### Ectopic expression of SNAIL enhances EMT in thyroid cancer cells

**Figure 4:**
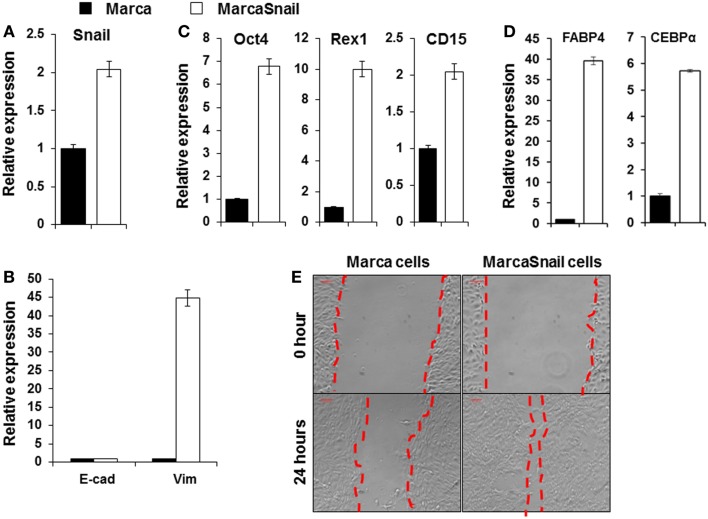
**Over-expression of Snail in BRAF^V600E^ thyroid cancer cells induced EMT**. Expression of Snail **(A)**; E-cadherin and vimentin **(B)**; and Oct4, Rex1, and CD15 **(C)** in thyroid cancer cells before and after transfection with the pCMV6-Snail vector. **(D)** Shows expression of adipocyte differentiation markers FABP4 and CEBPα in thyroid cancer cells before and after transfection with the pCMV6-Snail vector. Cells were analyzed after culture with adipocyte differentiation medium for 15 days. The results are expressed as mean ± SEM of three independent experiments with three parallels. **(E)** Migratory ability of thyroid cancer cells before and after transfection with the pCMV6-Snail vector.

Increased expression of Snail, Slug, Twist, ZEB1, and ZEB2 expression in thyroid cancers has been well demonstrated (34, 35). Snail appears to play a fundamental role in EMT and its expression is associated with invasiveness, metastases, tumor recurrence, and poor prognosis (36). Snail induction of EMT and CSC-like properties has been described in other cancers including breast and squamous cell carcinoma (37, 38). To study the function of Snail in thyroid cancer cells, we developed a thyroid cancer cell line (the Marca cell line) from the murine tumors initiated by oncogenic Braf (LSL-Braf(V600E)/TPO-Cre) and transfect the cells with pCMV6-SNAIL. Over-expression of Snail after transfection was demonstrated by qRT-PCR (Figure 4A). To test whether the expressed Snail was functional, we performed qRT-PCR for the EMT markers. Expression of the mesenchymal marker vimentin was markedly increased by over-expression of Snail compared to untransfected cells, as detected by qRT-PCR, but did not affect E-cadherin expression (Figure 4B). The stem cell markers Oct4, Rex1, and CD15 were also increased in cells over-expressing Snail compared to the control cells (Figure 4C). Furthermore, after being cultured in adipocyte differentiation medium, early adipocyte differentiation markers such as the fatty acid-binding protein 4 (FABP4) and CCAAT-enhancer-binding protein α (CEBP α) were markedly increased in cells over-expressing Snail compared to the control cells (Figure 4D). The results indicated the multipotential of cells over-expressing Snail. The same morphological changes of elongated and spindle-like shapes appeared to be further induced by such over-expression. In addition, the migration of cells across an artificial wound was increased with over-expression of Snail (Figure 4E). The results indicated that Snail over-expression induced EMT and the CSC-like phenotype in thyroid cancer cells and this enhanced the potential for cancer cell migration.

### Celastrol suppress TGF-β1 induced EMT

**Figure 5:**
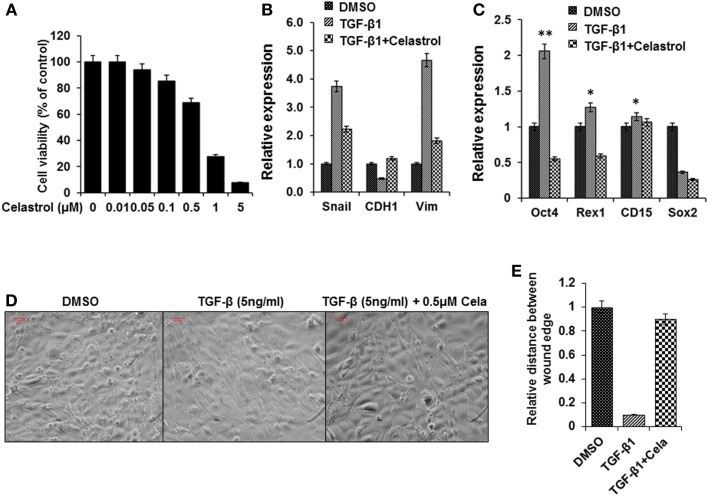
**Celastrol attenuated TGF-β1 induced EMT effects in thyroid cancer cells from BRAF^V600E^ mice**. **(A)** Cell viability of the thyroid cancer cells treated with celastrol for 48 h and cell viability was analyzed using a cell counting Kit-8. **(B,C)** Expression of EMT related markers Snail, E-cadherin, and vimentin **(B)** and Stem cell markers Oct4, Rex1, CD15, and Sox2 **(C)** in thyroid cells treated with TGF-β1 (5 ng/ml), or TGF-β1(5 ng/ml) and Celastrol (0.5μM), or the same concentration of vehicle (DMSO) for 72 h using qRT-PCR. The results are expressed as mean ± SEM of three independent experiments with three parallels. **p < 0.01 or *p < 0.05 indicated when thyroid cancer cells treated with TGF-β1 compared with cells treated with vehicle. **(D)** Morphological changes of thyroid cancer cells treated with TGF-β1 (5 ng/ml), or TGF-β1 (5 ng/ml) and Celastrol (0.5 μM), or the same concentration vehicle (DMSO) for 72 h. **(E)** The wound-healing results were scored in a double-blind method analyzing several areas of the wound after cells treated as above.

As described, there is evidence that TGF-β can induce EMT, which participates in the early stage of metastasis formation in a variety of epithelial tumors and this action is mediated by the Snail signaling pathway (15, 16). Celastrol, a compound used in traditional Chinese medicine, has been reported to suppress cancer cell migration by inhibiting TGF-β1 induced EMT (39). We found that celastrol (0.5 μM) did not reduce the cancer cell line viability (Figure 5A) but attenuated the effects of TGF-β1 on stem cell gene expression (Figures 5B,C). While treatment with TGF-β1 induced prominent morphological changes in the thyroid cancer cells, including elongated and spindle-like shapes, they were noticeable suppressed by co-treatment with celastrol (Figure 5D). Co-incubation with celastrol also reversed TGF-β1-mediated induction of cell migration. The results were scored using a double-blind method by analyzing several areas of the wound (Figure 5E). These findings showed that celastrol was able to inhibit the effects of TGF-β1 induced EMT in BRAF^V600E^ cancer cells.

## Discussion

In this study, using mice with a thyroid-specific knock-in of oncogenic Braf (LSL-Braf(V600E)/TPO-Cre), the thyroid epithelial cells became transformed and progressed to invasive carcinomas with a very short latency as shown by neoplastic transformation present at birth. These mice also become profoundly hypothyroid due to dysregulation of genes involved in thyroid hormone biosynthesis and consequently develop high TSH levels (22). We demonstrated the down-regulation of thyroid-specific markers (Tg and NIS) in their cancer tissue and this was reflected in isolated thyroid cancer cells derived from the tumors. We also found that expression of the epithelial marker E-cadherin was diminished. In contrast, there was up-regulation of stem cell markers (Oct4, Rex1, CD15, and Sox2) and the mesenchymal marker vimentin as evidenced by immunohistochemistry and RT-PCR. These data confirmed that EMT was induced in the thyroid carcinoma model of Braf^V600E^ mice (25) and, in particular, also demonstrated that thyroid cells acquire differentiated CSC-like properties by passing through EMT rather than deriving from resident stem cells which would not be activated by the TPO-dependent construct initiating the neoplastic response (9, 22, 26).

Epithelial to mesenchymal transition is a normal morphological event during embryonic development, tissue remodeling, and wound-healing but has also been shown to occur in neoplastic cells, especially in metastases (8). By the EMT processes, epithelial cells acquire fibroblast-like properties and exhibit reduced intercellular adhesion and increased motility providing cells undergoing EMT with cancer stem cell properties including enhanced invasive abilities and chemoresistance (9, 11). Such cells exhibit down-regulated E-cadherin and up-regulated vimentin expression as part of the acquisition of these changes, which have been shown to be induced by TGF-β1 (40) or increased expression of the transcription factor Snail (30, 37, 41).

Hence, EMT can be provoked by signals that cells receive from their microenvironment, such as TGF-β (15, 42) and several transcription factors have been implicated in the control of EMT in addition to Snail and including Slug, Twist, ZEB1/2, SIP1, and E12/E47 (29, 36, 43) all of which can be induced by TGF-β (18). In agreement, we found enhanced expression of the Snail family of EMT inducers (including Snail and Slug) and TGF-β1, TGF-β3, and TGF-βRII in the murine papillary thyroid carcinoma samples as shown in previous studies (13, 35, 44). Recent work has also demonstrated that thyroid cancer, which developed in BRAF^V600E^ mice were susceptible to TGF-β1 induced EMT (25) and BRAF^V600E^ promoted invasiveness of thyroid cancer cells by decreasing E-cadherin expression through a Snail-dependent mechanism (45). We also demonstrated that TGF-β1 or over-expression of Snail dramatically increased the mesenchymal marker vimentin and stem cell markers in thyroid cancer cells derived from BRAF^V600E^ mice. Furthermore, celastrol, a plant triterpene derived from the root of “Thunder of God Vine” (Tripterygium wilfordii Hook F.), exhibited anticancer potential and inhibited TGF-β1 induced EMT in the neoplastic cells (39, 46). Our findings demonstrated that celastrol attenuated TGF-β1 induced EMT and stemness related gene changes and cellular migration. However, the mechanism of this inhibition requires further clarification.

In conclusion, our results suggested that the thyroid papillary cancer of BRAF^V600E^ mice undergo EMT and dedifferentiate to acquire stem cell-like features. TGF-β1 treatment and over-expression of Snail in thyroid cancer cells derived from BRAF^V600E^ mice further reinforced EMT, while celastrol attenuated the TGF-β1 effects. Our findings provide strong evidence that thyroid cancer stemness in murine papillary cancer is derived via the EMT process and that thyroid CSCs are not derived from mutated thyroid stem cells. Hence, these data support the stochastic model where every malignant thyroid cancer cell has the potential to act as a CSC rather than CSCs being a biologically distinct subset within the malignant cell population.

## Conflict of Interest Statement

The authors declare that the research was conducted in the absence of any commercial or financial relationships that could be construed as a potential conflict of interest.

## References

[1] Lin RY Thyroid cancer stem cells. Nat Rev Endocrinol (2011) 7:609–16 10.1038/nrendo.2011.127 21788969

[2] Hoshi N Kusakabe T Taylor BJ Kimura S Side population cells in the mouse thyroid exhibit stem/progenitor cell-like characteristics. Endocrinology (2007) 148:4251–8 10.1210/en.2006-0490 17584961 PMC2582754

[3] Thomas T Nowka K Lan L Derwahl M Expression of endoderm stem cell markers: evidence for the presence of adult stem cells in human thyroid glands. Thyroid (2006) 16:537–44 10.1089/thy.2006.16.537 16839255

[4] Lan L Cui D Nowka K Derwahl M Stem cells derived from goiters in adults form spheres in response to intense growth stimulation and require thyrotropin for differentiation into thyrocytes. J Clin Endocrinol Metab (2007) 92:3681–8 10.1210/jc.2007-0281 17609303

[5] Fierabracci A Puglisi MA Giuliani L Mattarocci S Gallinella-Muzi M Identification of an adult stem/progenitor cell-like population in the human thyroid. J Endocrinol (2008) 198:471–87 10.1677/JOE-07-0552 18550786

[6] Lan L Luo Y Cui D Shi BY Deng W Huo LL Epithelial-mesenchymal transition triggers cancer stem cell generation in human thyroid cancer cells. Int J Oncol (2013) 43:113–20 10.3892/ijo.2013.1913 23604232

[7] Ma R Minsky N Morshed SA Davies TF Stemness in human thyroid cancers and derived cell lines: the role of asymmetrically dividing cancer stem cells resistant to chemotherapy. J Clin Endocrinol Metab (2014) 99:E400–9 10.1210/jc.2013-3545 24823711 PMC3942234

[8] Thiery JP Acloque H Huang RY Nieto MA Epithelial-mesenchymal transitions in development and disease. Cell (2009) 139:871–90 10.1016/j.cell.2009.11.007 19945376

[9] Mani SA Guo W Liao MJ Eaton EN Ayyanan A Zhou AY The epithelial-mesenchymal transition generates cells with properties of stem cells. Cell (2008) 133:704–15 10.1016/j.cell.2008.03.027 18485877 PMC2728032

[10] Hollier BG Evans K Mani SA The epithelial-to-mesenchymal transition and cancer stem cells: a coalition against cancer therapies. J Mammary Gland Biol Neoplasia (2009) 14:29–43 10.1007/s10911-009-9110-3 19242781

[11] Polyak K Weinberg RA Transitions between epithelial and mesenchymal states: acquisition of malignant and stem cell traits. Nat Rev Cancer (2009) 9:265–73 10.1038/nrc2620 19262571

[12] Kalluri R Weinberg RA The basics of epithelial-mesenchymal transition. J Clin Invest (2009) 119:1420–8 10.1172/JCI39104 19487818 PMC2689101

[13] Buehler D Hardin H Shan W Montemayor-Garcia C Rush PS Asioli S Expression of epithelial-mesenchymal transition regulators SNAI2 and TWIST1 in thyroid carcinomas. Mod Pathol (2013) 26:54–61 10.1038/modpathol.2012.137 22899291 PMC3559085

[14] Vasko V Espinosa AV Scouten W He H Auer H Liyanarachchi S Gene expression and functional evidence of epithelial-to-mesenchymal transition in papillary thyroid carcinoma invasion. Proc Natl Acad Sci U S A (2007) 104:2803–8 10.1073/pnas.0610733104 17296934 PMC1815262

[15] Massague J Blain SW Lo RS TGFbeta signaling in growth control, cancer, and heritable disorders. Cell (2000) 103:295–309 10.1016/S0092-8674(00)00121-5 11057902

[16] Tirino V Camerlingo R Bifulco K Irollo E Montella R Paino F TGF-beta1 exposure induces epithelial to mesenchymal transition both in CSCs and non-CSCs of the A549 cell line, leading to an increase of migration ability in the CD133+ A549 cell fraction. Cell Death Dis (2013) 4:e620 10.1038/cddis.2013.144 23640462 PMC3674353

[17] Batlle E Sancho E Franci C Dominguez D Monfar M Baulida J The transcription factor snail is a repressor of E-cadherin gene expression in epithelial tumour cells. Nat Cell Biol (2000) 2:84–9 10.1038/35000034 10655587

[18] Cano A Perez-Moreno MA Rodrigo I Locascio A Blanco MJ del Barrio MG The transcription factor snail controls epithelial-mesenchymal transitions by repressing E-cadherin expression. Nat Cell Biol (2000) 2:76–83 10.1038/35010506 10655586

[19] Hajra KM Chen DY Fearon ER The SLUG zinc-finger protein represses E-cadherin in breast cancer. Cancer Res (2002) 62:1613–8 11912130

[20] Yang J Mani SA Donaher JL Ramaswamy S Itzykson RA Come C Twist, a master regulator of morphogenesis, plays an essential role in tumor metastasis. Cell (2004) 117:927–39 10.1016/j.cell.2004.06.006 15210113

[21] Ponnusamy MP Lakshmanan I Jain M Das S Chakraborty S Dey P MUC4 mucin-induced epithelial to mesenchymal transition: a novel mechanism for metastasis of human ovarian cancer cells. Oncogene (2010) 29:5741–54 10.1038/onc.2010.309 20697346 PMC3005772

[22] Franco AT Malaguarnera R Refetoff S Liao XH Lundsmith E Kimura S Thyrotrophin receptor signaling dependence of Braf-induced thyroid tumor initiation in mice. Proc Natl Acad Sci U S A (2011) 108:1615–20 10.1073/pnas.1015557108 21220306 PMC3029699

[23] Mercer K Giblett S Green S Lloyd D DaRocha Dias S Plumb M Expression of endogenous oncogenic V600EB-raf induces proliferation and developmental defects in mice and transformation of primary fibroblasts. Cancer Res (2005) 65:11493–500 10.1158/0008-5472.CAN-05-2211 16357158 PMC2640458

[24] Kusakabe T Kawaguchi A Kawaguchi R Feigenbaum L Kimura S Thyrocyte-specific expression of Cre recombinase in transgenic mice. Genesis (2004) 39:212–6 10.1002/gene.20043 15282748

[25] Knauf JA Sartor MA Medvedovic M Lundsmith E Ryder M Salzano M Progression of BRAF-induced thyroid cancer is associated with epithelial-mesenchymal transition requiring concomitant MAP kinase and TGFbeta signaling. Oncogene (2011) 30:3153–62 10.1038/onc.2011.44 21383698 PMC3136543

[26] Kong D Banerjee S Ahmad A Li Y Wang Z Sethi S Epithelial to mesenchymal transition is mechanistically linked with stem cell signatures in prostate cancer cells. PLoS One (2010) 5:e12445 10.1371/journal.pone.0012445 20805998 PMC2929211

[27] Pardali K Moustakas A Actions of TGF-beta as tumor suppressor and pro-metastatic factor in human cancer. Biochim Biophys Acta (2007) 1775:21–62 16904831 10.1016/j.bbcan.2006.06.004

[28] Ikushima H Miyazono K TGFbeta signalling: a complex web in cancer progression. Nat Rev Cancer (2010) 10:415–24 10.1038/nrc2853 20495575

[29] Moustakas A Heldin CH Signaling networks guiding epithelial-mesenchymal transitions during embryogenesis and cancer progression. Cancer Sci (2007) 98:1512–20 10.1111/j.1349-7006.2007.00550.x 17645776 PMC11158989

[30] Naber HP Drabsch Y Snaar-Jagalska BE ten Dijke P van Laar T Snail and Slug, key regulators of TGF-beta-induced EMT, are sufficient for the induction of single-cell invasion. Biochem Biophys Res Commun (2013) 435:58–63 10.1016/j.bbrc.2013.04.037 23618854

[31] Vincent T Neve EP Johnson JR Kukalev A Rojo F Albanell J A SNAIL1-SMAD3/4 transcriptional repressor complex promotes TGF-beta mediated epithelial-mesenchymal transition. Nat Cell Biol (2009) 11:943–50 10.1038/ncb1905 19597490 PMC3769970

[32] Becker KF Rosivatz E Blechschmidt K Kremmer E Sarbia M Hofler H Analysis of the E-cadherin repressor Snail in primary human cancers. Cells Tissues Organs (2007) 185:204–12 10.1159/000101321 17587826

[33] Come C Magnino F Bibeau F De Santa Barbara P Becker KF Theillet C Snail and slug play distinct roles during breast carcinoma progression. Clin Cancer Res (2006) 12:5395–402 10.1158/1078-0432.CCR-06-0478 17000672

[34] Vasko V Bauer AJ Tuttle RM Francis GL Papillary and follicular thyroid cancers in children. Endocr Dev (2007) 10:140–72 10.1159/000106825 17684395

[35] Hardy RG Vicente-Duenas C Gonzalez-Herrero I Anderson C Flores T Hughes S Snail family transcription factors are implicated in thyroid carcinogenesis. Am J Pathol (2007) 171:1037–46 10.2353/ajpath.2007.061211 17724139 PMC1959496

[36] Peinado H Olmeda D Cano A Snail, Zeb and bHLH factors in tumour progression: an alliance against the epithelial phenotype? Nat Rev Cancer (2007) 7:415–28 10.1038/nrc2131 17508028

[37] Fan F Samuel S Evans KW Lu J Xia L Zhou Y Overexpression of snail induces epithelial-mesenchymal transition and a cancer stem cell-like phenotype in human colorectal cancer cells. Cancer Med (2012) 1:5–16 10.1002/cam4.4 23342249 PMC3544430

[38] Zhu LF Hu Y Yang CC Xu XH Ning TY Wang ZL Snail overexpression induces an epithelial to mesenchymal transition and cancer stem cell-like properties in SCC9 cells. Lab Invest (2012) 92:744–52 10.1038/labinvest.2012.8 22349639

[39] Kang H Lee M Jang SW Celastrol inhibits TGF-beta1-induced epithelial-mesenchymal transition by inhibiting Snail and regulating E-cadherin expression. Biochem Biophys Res Commun (2013) 437:550–6 10.1016/j.bbrc.2013.06.113 23850675

[40] Wu Y Fu Y Zheng L Lin G Ma J Lou J Nutlin-3 inhibits epithelial-mesenchymal transition by interfering with canonical transforming growth factor-beta1-Smad-Snail/Slug axis. Cancer Lett (2014) 342:82–91 10.1016/j.canlet.2013.08.039 24001610

[41] Dang H Ding W Emerson D Rountree CB Snail1 induces epithelial-to-mesenchymal transition and tumor initiating stem cell characteristics. BMC Cancer (2011) 11:396 10.1186/1471-2407-11-396 21929801 PMC3189192

[42] Christofori G New signals from the invasive front. Nature (2006) 441:444–50 10.1038/nature04872 16724056

[43] Nieto MA The snail superfamily of zinc-finger transcription factors. Nat Rev Mol Cell Biol (2002) 3:155–66 10.1038/nrm757 11994736

[44] Salerno P Garcia-Rostan G Piccinin S Bencivenga TC Di Maro G Doglioni C TWIST1 plays a pleiotropic role in determining the anaplastic thyroid cancer phenotype. J Clin Endocrinol Metab (2011) 96:E772–81 10.1210/jc.2010-1182 21389145

[45] Baquero P Sanchez-Hernandez I Jimenez-Mora E Orgaz JL Jimenez B Chiloeches A (V600E)BRAF promotes invasiveness of thyroid cancer cells by decreasing E-cadherin expression through a Snail-dependent mechanism. Cancer Lett (2013) 335:232–41 10.1016/j.canlet.2013.02.033 23435375

[46] Yang H Chen D Cui QC Yuan X Dou QP Celastrol, a triterpene extracted from the Chinese “Thunder of God Vine,” is a potent proteasome inhibitor and suppresses human prostate cancer growth in nude mice. Cancer Res (2006) 66:4758–65 10.1158/0008-5472.CAN-05-4529 16651429

